# Refractive error after phacoemulsification combined with intraocular lens implantation in primary angle-closure glaucoma: a multifactorial analysis of biometric parameters and surgical strategies

**DOI:** 10.3389/fcell.2025.1654719

**Published:** 2025-08-22

**Authors:** Yong Liu, Kuanrong Dang, Di Gong, Simin Deng, Yin Deng, Junhong Guo, Xiaoli Shen, Jiantao Wang

**Affiliations:** ^1^ Jinan University, Guangzhou, Guangdong, China; ^2^ Shenzhen Eye Hospital, Shenzhen Eye Medical Center, Southern Medical University, Guangzhou, China; ^3^ The Second Clinical Medical College (Shenzhen People’s Hospital) of Jinan University, Shenzhen, China

**Keywords:** primary angle-closure glaucoma, cataract surgery, refractive error, prediction error, postoperative refractive outcomes

## Abstract

**Purpose:**

This study aims to explore the factors influencing refractive error following Phacoemulsification combined with intraocular lens implantation (PE + IOL) in patients with primary angle-closure glaucoma (PACG), providing a theoretical basis for preoperative consultation and IOL power selection in clinical practice.

**Methods:**

A retrospective analysis was conducted on 404 PACG patients from Shenzhen Eye Hospital between 2019 and 2024. Preoperative ocular biometric parameters and combined surgical approaches were evaluated using Spearman correlation, multinomial logistic regression, and receiver operating characteristic (ROC) curve analysis.

**Results:**

Axial length (AL), lens thickness (LT), and white-to-white distance (WTW) were key predictive factors for prediction error and postoperative refractive outcomes. AL > 22.56 mm (AUC = 0.692) and LT > 5.055 mm (AUC = 0.633) increased the risk of myopic shift (MS), while AL < 22.25 mm (AUC = 0.604) and WTW <11.55 mm (OR = 2.209, *P* = 0.001) were associated with hyperopic shift (HS). The axial length/corneal radius (AL/CR) ratio >2.986 further indicated a higher risk of MS (AUC = 0.639) postoperatively. Among patients who underwent PE + IOL combined with capsular tension ring, the proportion of HS was significantly higher (Z value = +2.95).

**Conclusion:**

The unique anatomical characteristics of PACG patients are key contributors to postoperative refractive instability. Preoperative assessment for PE + IOL surgery should comprehensively evaluate AL, LT, WTW, and AL/CR ratio. When combined with other surgical approaches to control intraocular pressure, IOL power should be adjusted according to these thresholds to ensure postoperative refractive stability.

## Introduction

Primary angle-closure glaucoma (PACG) is one of the most vision-threatening types of glaucoma, with a blindness rate as high as 27.0% (George et al., 2022). PACG is more prevalent in Asian populations than in other regions, accounting for over half of the global cases (He et al., 2006; Shan et al., 2024; Tham et al., 2014; Xu et al., 2025). Its hallmark is the progressive synechiae of the anterior chamber angle, leading to the obstruction of aqueous humor drainage pathways and a subsequent increase in intraocular pressure (IOP).

Currently, lowering IOP is the only proven method to prevent or delay the progression of glaucoma. Several studies have shown that in patients with mild-moderate PACG, phacoemulsification combined with intraocular lens implantation (PE + IOL) can reduce IOP, improve vision (Hirasawa et al., 2018; Zhang et al., 2016), increase anterior chamber depth and width, and decrease the need for postoperative anti-glaucoma medications (Angmo et al., 2019; Ghadamzadeh et al., 2022; Nonaka et al., 2006). However, surgical outcomes are closely related to the stage of glaucoma. Song et al. (2025) suggested that for patients with advanced PACG (with or without coexisting cataract), a surgical approach involving PE + IOL combined with goniosynechialysis (GSL) and gonioscopy-assisted transluminal trabeculotomy (GATT) may be considered. Notably, a meta-analysis showed that minimally invasive glaucoma surgery (MIGS) based on PE + IOL with or without GSL plus trabecular meshwork-Schlemm canal procedures can effectively lower IOP and medication burden in PACG patients while maintaining a favorable safety profile, with the iStent group showing the lowest complication rate (Fang et al., 2025). However, patients undergoing PE + IOL with iStent surgery were at a higher risk of developing cystoid macular edema and anterior uveitis compared to phacoemulsification alone (Issa et al., 2025). Furthermore, clear lens extraction (CLE) is also effective in treating young PACG patients, although postoperative refractive predictability is less reliable (Day et al., 2018). Inappropriate selection of intraocular lens (IOL) power calculation formula (Lu et al., 2022), corneal edema affecting the accuracy of biometrics, changes in ocular anatomy, and displacement of the lens capsule after cataract surgery may all contribute to inaccurate postoperative refractive predictions in PACG patients (Li et al., 2021).

Regarding IOL power calculation formulas, studies have shown that the Barrett Universal II formula is commonly used to predict IOL power (Ji et al., 2024). However, a recent network meta-analysis incorporating eight IOL power calculation formulas (Lu et al., 2022) found no significant correlation between postoperative refractive status and IOL power calculation formulas in ACG patients undergoing PE + IOL surgery. Additionally, multiple studies have found that patients with preoperative shallow anterior chambers, short axial length, and high IOP are more likely to experience significant refractive prediction errors (Ji et al., 2024; Li et al., 2021; Shin et al., 2023). These characteristics are specific anatomical features of PACG patients (Kang et al., 2009). Therefore, despite the significant effects of PE + IOL surgery in improving visual function and controlling IOP (Zaidi et al., 2007), postoperative refractive error (RE) remains a major issue in the clinical management of PACG. Clinical observations suggest that biometric parameters, such as anterior chamber depth (ACD), axial length (AL), and lens thickness (LT), have certain predictive effects on postoperative refractive status. Thus, this study aims to analyze the relevant influencing factors of postoperative refractive error by examining ocular biometric parameters, providing theoretical support for preoperative consultation and IOL selection.

## Materials and methods

### Study design

This study is a retrospective analysis aimed at investigating the factors influencing refractive errors in PACG patients following PE + IOL surgery and predicting the postoperative refractive outcomes in this group. Data for this study were extracted from the electronic medical records of Shenzhen Eye Hospital, covering the period from June 2019 to June 2024, including clinical data and relevant preoperative and postoperative examination data of the patients. All patients provided informed consent before surgery, fully acknowledging the potential risk of postoperative refractive errors. This study was approved by the Ethics Committee of Shenzhen Eye Hospital (Ethical Approval No. 2024KYPJ057) and adheres to the guidelines of the Helsinki Declaration and applicable ethical regulations, ensuring the research process conforms to ethical standards.

### Study population

**FIGURE 1:**
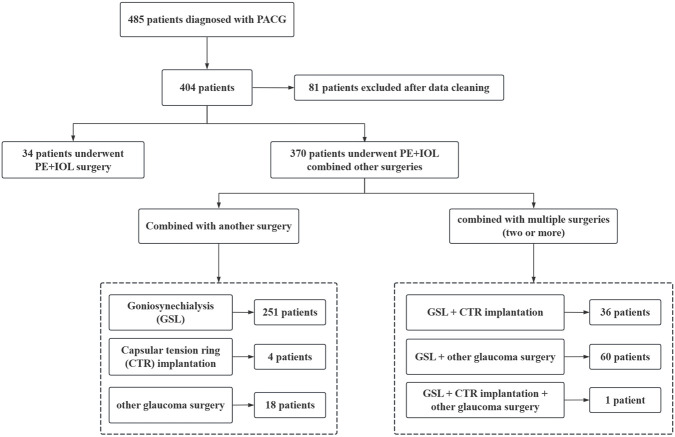
Distribution of clinical features in the included patients.

The participants in this study are patients diagnosed with PACG (≥180° iridotrabecular contact with peripheral anterior synechiae, elevated intraocular pressure, and glaucomatous optic neuropathy) who underwent PE + IOL surgery, with the distribution of these patients, including those who received combined procedures, shown in Figure 1. Inclusion criteria: 1) Diagnosis of mild-moderate chronic PACG (Foster et al., 2002); 2) Age over 40 years; 3) Diagnosis of age-related cataract; 4) Complete preoperative and postoperative clinical data with a minimum postoperative follow-up of 6 months; 5) IOL power calculated using the Barrett Universal II formula before surgery; 6) A smooth surgery without major intraoperative or postoperative complications (e.g., posterior capsule rupture, infectious endophthalmitis, corneal endothelial decompensation, etc.); 7) Implantation of a monofocal IOL. Exclusion criteria: 1) Patients with other ocular diseases that may affect postoperative refractive outcomes (e.g., corneal diseases, fundus diseases, or retinal diseases, etc.); 2) History of acute angle-closure glaucoma attack, defined as a sudden and marked increase in intraocular pressure accompanied by visual deterioration, eye pain, corneal edema, and a mid-dilated pupil. All surgeries were performed by experienced surgeons (X.S. and J.W.) using the Infiniti phacoemulsification machine (Alcon, United States). Thirty minutes before surgery, compound tropicamide eye drops were used for full mydriasis, and 0.4% oxybuprocaine hydrochloride eye drops were used to confirm local anesthesia. After making the main and side-port incisions, a continuous circular capsulorhexis with a diameter of approximately 5.5 mm was performed. The subsequent steps included hydrodissection, hydrodelamination, lens phacoemulsification, cortical aspiration, polishing of both anterior and posterior capsules, and implantation of the IOL into the capsule, with adjustments made to center the IOL and ensure the posterior capsule remained wrinkle-free. Finally, watertight closure of clear corneal incisions was performed to stabilize the anterior chamber.

### Study variables and measurement methods

The research data will be collected by reviewing the patients’ electronic medical records, including basic information, ocular examination results, and preoperative and postoperative clinical data. These data include basic information such as age, sex, body mass index (BMI), past medical history (e.g., hypertension, diabetes), previous glaucoma surgery, refractive data (including preoperative and postoperative refractive measurements), and preoperative target refractive power.

All subjects underwent a comprehensive ocular examination, including optometry, IOP measurement following the use of anti-glaucoma drugs, corneal endothelial examination, and ocular biometrics using the IOL-Master 700 (Carl Zeiss Medical, Dublin, Ireland). The IOL-Master 700, operated by trained professionals, was used to measure AL, corneal curvature, ACD, central corneal thickness (CCT), and LT. The Barrett Universal II formula was used to calculate IOL power and predict postoperative refractive error. Best-corrected visual acuity (BCVA) and IOP measurements were taken after PE + IOL surgery.

∆IOP is defined as the difference between preoperative and postoperative intraocular pressures. The postoperative prediction error is calculated by subtracting the postoperative spherical equivalent (SE) predicted by the Barrett Universal II formula from the actual postoperative SE. Actual postoperative refractive power is defined as the spherical equivalent refractive power measured 3–6 months following PE + IOL surgery. Accurate IOL power prediction (i.e., postoperative emmetropia) is defined as PE between −0.50 diopters (D) and 0.50D. In this study, hyperopic shift (HS) is defined as PE > 0.50 D, and myopic shift (MS) is defined as PE < −0.50 D. These categories were used as the dependent variable in multinomial logistic regression analysis to evaluate the association between ocular parameters and postoperative refractive outcomes.

### Statistical analysis

All data were analyzed using R software (version 4.2.3). First, descriptive statistics were performed on the basic characteristics and variables of the patients. Continuous variables were expressed as mean ± standard deviation (Mean ± SD) or median (interquartile range), and categorical variables as frequencies. Comparisons between groups for continuous variables were performed using t-tests or Mann-Whitney U tests, while categorical variables were compared using chi-square tests or Fisher’s exact tests. All statistical analyses were performed using two-tailed tests, and differences were considered statistically significant at *P* < 0.05. To assess the factors influencing postoperative prediction error, Model 1 (full model) was used, which included all variables with *P* < 0.10. This model aimed to evaluate the impact of these variables on prediction error. To further simplify, Model 2 (optimal model from stepwise) was adopted, which selected the most significant variables from Model 1 using stepwise regression. The variables included in the final model were ACD, AL, CCT, LT, white-to-white distance (WTW), postoperative BCVA, and combined with capsular tension ring (CTR) implantation. The purpose of this model was to improve predictive power by selecting only the most relevant variables. Regression analysis was conducted after adjusting for baseline characteristics such as age, sex, and the axial length/corneal radius (AL/CR) ratio. The two models were compared using likelihood ratio *χ* ^*2*^ tests to evaluate whether there were significant differences between them. The analysis revealed that the AIC for Model 1 and Model 2 were 747.459 and 736.655, respectively, with a likelihood ratio *χ* ^*2*^ of 13.196 and *P*-value of 0.355, indicating no significant difference between the two models.

## Results

### Demographics and clinical characteristics

This study included 404 patients, with the demographic data shown in Table 1. The average age of the study population was 65.72 ± 8.43 years, with a majority of females (74.0%, 299/404). In terms of health conditions, approximately 128 patients had hypertension, while diabetes was less prevalent, affecting 48 patients. Additionally, 277 patients had not undergone previous glaucoma surgery, while 127 had received such surgery. Regarding the treatment plan, 348 patients underwent combined GSL surgery, while only 41 patients underwent PE + IOL combined with CTR implantation.

**TABLE 1:** Clinical characteristics of the study population.

Variables	Mean ± SD or N
Age, years	65.72 ± 8.43
ACD, mm	2.25 ± 0.33
AL, mm	22.65 ± 0.84
AL/CR	2.99 ± 0.09
BMI, kg/m^2^	23.86 ± 3.32
CCT, mm	551.30 ± 46.70
CECD, N/mm^2^	2,438.05 ± 456.40
LT, mm	5.00 ± 0.37
Preoperative BCVA	0.47 ± 0.51
Postoperative BCVA	0.16 ± 0.33
Preoperative IOP, mmHg	19.42 ± 10.08
Postoperative IOP, mmHg	13.66 ± 3.63
Prediction error, Diopter	0.17 ± 0.74
∆IOP, mmHg	5.75 ± 10.30
WTW, mm	11.48 ± 0.52
P, mm	3.77 ± 1.32
K1, Diopter	44.06 ± 1.58
K2, Diopter	44.98 ± 1.57
Sex (Male/Female)	105/299
Hypertension (No/Yes)	276/128
Diabetes (No/Yes)	356/48
Combined with GSL (No/Yes)	56/348
Combined with CTR implantation (No/Yes)	363/41
Combined with other glaucoma surgery (No/Yes)	325/79
Previous glaucoma surgery (No/Yes)	277/127

ACD, Anterior chamber depth; AL, Axial length; AL/CR, Axial length/corneal radius; BCVA, Best-corrected visual acuity; BMI, Body mass index; CCT, Central corneal thickness; CECD, Corneal endothelial cell density; CTR, Capsular tension ring; GSL, Goniosynechialysis; IOP, Intraocular pressure; K1, Flat Axis Curvature; K2, Steep Axis Curvature; LT, Lens thickness; P, Pupil diameter; WTW, White-to-White Distance.

Regarding ocular biometric parameters, the average ACD was 2.25 ± 0.33 mm, AL was 22.65 ± 0.84 mm, LT was 5.00 ± 0.37 mm, and AL/CR ratio was 2.99 ± 0.09. The mean IOP reduction from preoperative to postoperative values was 5.75 ± 10.30 mmHg, and the mean prediction error was 0.17 ± 0.74 D.

### Correlation analysis of prediction error

**FIGURE 2:**
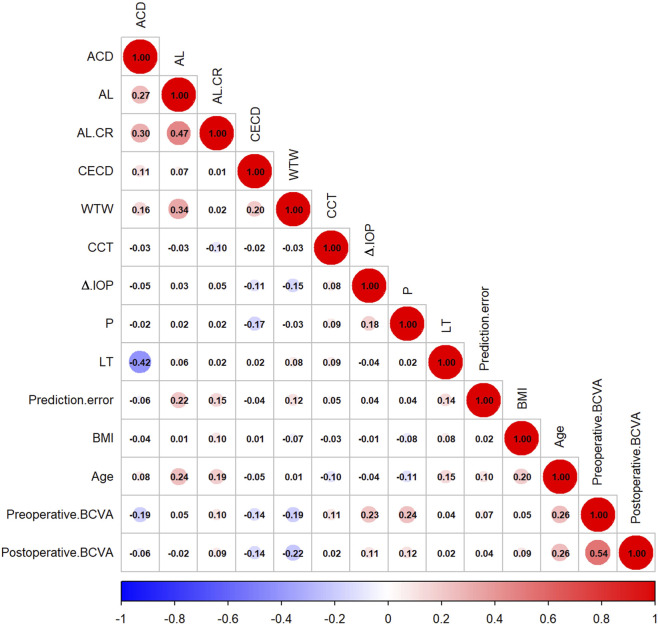
Correlation matrix of predictive error with different variables.

Table 2 shows the correlation analysis between different variables and prediction error. Among continuous variables, Spearman’s correlation analysis (Figure 2) showed that age (*ρ* = 0.100, *P* = 0.044), AL (*ρ* = 0.216, *P* < 0.001), AL/CR ratio (*ρ* = 0.153, *P* = 0.002), LT (*ρ* = 0.138, *P* = 0.006), and WTW (*ρ* = 0.115, *P* = 0.021) were significantly positively correlated with prediction error. In contrast, variables such as BMI, CCT, and corneal endothelial cell density (CECD) were not significantly correlated (*P* > 0.05).

**TABLE 2:** Spearman correlation analysis between different variables and prediction error.

Variables	*ρ*	*P*
Age, years	0.100	0.044
ACD, mm	−0.065	0.195
AL, mm	0.216	<0.001
AL/CR	0.153	0.002
BMI, kg/m^2^	0.017	0.727
CCT, mm	0.054	0.281
CECD, N/mm^2^	−0.035	0.481
LT, mm	0.138	0.006
Preoperative BCVA	0.071	0.154
Postoperative BCVA	0.036	0.466
Preoperative IOP, mmHg	0.022	0.665
Postoperative IOP, mmHg	−0.088	0.077
∆IOP, mmHg	0.036	0.466
WTW, mm	0.115	0.021
P, mm	0.035	0.479
K1, Diopter	−0.052	0.297
K2, Diopter	−0.095	0.056

ACD, Anterior chamber depth; AL, Axial length; AL/CR, Axial length/corneal radius; BCVA, Best-corrected visual acuity; BMI, Body mass index; CCT, Central corneal thickness; CECD, Corneal endothelial cell density; IOP, Intraocular pressure; K1, Flat Axis Curvature; K2, Steep Axis Curvature; LT, Lens thickness; P, Pupil diameter; WTW, White-to-White Distance.

Table 3 shows the distribution of postoperative refractive outcomes across different clinical variables. Regarding sex, there was a statistically significant difference among the three groups (*χ* ^*2*^ = 7.175, *P* = 0.028). The proportion of females was highest in the myopic shift group (88.1%), while males accounted for the lowest proportion in this group (11.9%). Combined with CTR implantation showed a significant difference (*χ* ^*2*^ = 8.822, *P* = 0.012), with the highest rate observed in the hyperopic shift group (16.4%) and the lowest in the myopic shift group (3.4%). The distribution of diabetes among the groups approached statistical significance (*χ* ^*2*^ = 5.122, *P* = 0.077), with the lowest prevalence observed in the myopic shift group (3.4%), compared to 12.6% in the emmetropia group and 14.8% in the hyperopic shift group. Other variables, including hypertension, combined with GSL, combined with other glaucoma surgery, and previous glaucoma surgery, did not show significant differences among the groups (*P* > 0.05).

**TABLE 3:** Distribution of postoperative refractive outcomes across different variables.

Variables, N (%)	Emmetropia	Hyperopic shift	Myopic shift	*χ* ^*2*^	*P*
Sex				7.175	0.028
Male	63 (28.3%)	35 (28.7%)	7 (11.9%)		
Female	160 (71.7%)	87 (71.3%)	52 (88.1%)		
Hypertension				1.314	0.518
No	151 (67.7%)	81 (66.4%)	44 (74.6%)		
Yes	72 (32.3%)	41 (33.6%)	15 (25.4%)		
Diabetes				5.122	0.077
No	195 (87.4%)	104 (85.2%)	57 (96.6%)		
Yes	28 (12.6%)	18 (14.8%)	2 (3.4%)		
Combined with GSL				1.504	0.471
No	34 (15.2%)	13 (10.7%)	9 (15.3%)		
Yes	189 (84.8%)	109 (89.3%)	50 (84.7%)		
Combined with CTR implantation				8.822	0.012
No	204 (91.5%)	102 (83.6%)	57 (96.6%)		
Yes	19 (8.5%)	20 (16.4%)	2 (3.4%)		
Combined with other glaucoma surgery				4.699	0.095
No	174 (78.0%)	106 (86.9%)	45 (76.3%)		
Yes	49 (22.0%)	16 (13.1%)	14 (23.7%)		
Previous glaucoma surgery				0.021	0.990
No	153 (68.6%)	84 (68.9%)	40 (67.8%)		
Yes	70 (31.4%)	38 (31.1%)	19 (32.2%)		

CTR, Capsular tension ring; GSL, goniosynechialysis.

### Univariate and multivariate analysis

Univariate analysis revealed that age (*F* = 3.325, *P* = 0.037), AL (*F* = 26.632, *P* < 0.001), AL/CR ratio (*F* = 13.224, *P* = 0.001), LT (*F* = 6.334, *P* = 0.002), preoperative best corrected visual acuity (Pre-BCVA, *F* = 7.986, *P* = 0.018), postoperative best corrected visual acuity (Post-BCVA, *F* = 7.110, *P* = 0.029), and WTW (*F* = 7.397, *P* = 0.025) were significantly correlated with postoperative refractive outcomes.

The likelihood ratio test revealed that the stepwise regression model (Model 2) showed no significant difference in predictive performance compared to the full model (Model 1) (*χ* ^*2*^ = 13.196, *P* = 0.355), suggesting that key variables such as AL, LT, and WTW were sufficient to support the predictive framework. Table 4 shows that AL was significantly associated with a reduced risk of MS in both Model 1 and Model 2 (Model 1, OR = 0.642, 95% CI: 0.445 to 0.924, *P* = 0.017; Model 2, OR = 0.568, 95% CI: 0.407 to 0.794, *P* = 0.001). LT also showed a trend of reducing the risk of MS in multinomial logistic regression (Model 1, OR = 0.203, 95% CI: 0.072 to 0.569, *P* = 0.002; Model 2, OR = 0.149, 95% CI: 0.056 to 0.399, *P* < 0.001).

**TABLE 4:** Univariate and multivariate analysis of postoperative refractive outcomes.

Variable	Univariate analysis		Multivariable analysis (model 1)				Multivariable analysis (model 2)			
	*F/H/χ* ^*2*^	*P*	OR (95% CI)	*P*_1	OR (95% CI)	*P*_2	OR (95% CI)	*P*_1	OR (95% CI)	*P*_2
^a^Age	3.325	0.037	1.002 (0.972, 1.034)	0.882	0.977 (0.939, 1.017)	0.264				
^b^ACD	4.981	0.083	0.357 (0.139, 0.914)	0.032	0.408 (0.113, 1.471)	0.171	0.363 (0.154, 0.858)	0.021	0.294 (0.086, 1.012)	0.052
^b^AL	26.632	<0.001	1.212 (0.865, 1.699)	0.263	0.642 (0.445, 0.924)	0.017	1.389 (1.079, 1.788)	0.011	0.568 (0.407, 0.794)	0.001
^b^AL/CR	13.224	0.001	7.960 (0.893, 70.969)	0.063	0.216 (0.072, 0.647)	0.006				
^b^BMI	0.942	0.625								
^b^CCT	5.576	0.062	1.006 (1.001, 1.011)	0.019	1.006 (0.999, 1.012)	0.093	1.007 (1.002, 1.012)	0.005	1.007 (1.001, 1.013)	0.018
^b^CECD	0.073	0.964								
^a^LT	6.334	0.002	0.666 (0.323, 1.374)	0.271	0.203 (0.072, 0.569)	0.002	0.717 (0.364, 1.414)	0.337	0.149 (0.056, 0.399)	<0.001
^b^Pre-BCVA	7.986	0.018	1.254 (0.692, 2.273)	0.456	1.237 (0.600, 2.551)	0.564				
^b^Post-BCVA	7.110	0.029	2.897 (1.042, 8.053)	0.041	4.015 (1.194, 13.505)	0.025	3.538 (1.475, 8.485)	0.005	4.364 (1.664, 11.447)	0.003
^b^Pre-IOP	3.532	0.171								
^b^Post-IOP	2.242	0.326								
^b^&^b^8710;IOP	1.737	0.420								
^b^P	2.425	0.298								
^a^WTW	7.397	0.025	2.209 (1.399, 3.488)	0.001	1.177 (0.667, 2.076)	0.573	2.065 (1.327, 3.212)	0.001	1.210 (0.710, 2.061)	0.483
^b^K1	3.645	0.162								
^b^K2	4.108	0.128								
^c^Sex	7.175	0.028	0.974 (0.564, 1.681)	0.923	1.435 (0.582, 3.539)	0.433				
^c^Hypertension	1.314	0.518								
^c^Diabetes	5.122	0.077	1.215 (0.606, 2.438)	0.583	0.314 (0.070, 1.415)	0.131				
^c^Combined with GSL	1.504	0.471								
^c^Combined with other glaucoma surgery	4.699	0.095	0.601 (0.312, 1.159)	0.129	1.095 (0.513, 2.335)	0.814				
^c^Previous glaucoma surgery	0.021	0.990								
^c^Combined with CTR implantation	8.822	0.012	1.526 (0.713, 3.262)	0.276	0.373 (0.078, 1.782)	0.216	1.723 (0.821, 3.613)	0.150	0.361 (0.076, 1.709)	0.199

^a^ ANOVA.

^b^ Kruskal–Wallis test.

^c^ Chi-square test; ACD, Anterior chamber depth; AL, Axial length; AL/CR, Axial length/corneal radius; BCVA, Best-corrected visual acuity; BMI, Body mass index; CCT, Central corneal thickness; CECD, Corneal endothelial cell density; CTR, Capsular tension ring; GSL, Goniosynechialysis; IOP, Intraocular pressure; K1, Flat Axis Curvature; K2, Steep Axis Curvature; LT, Lens thickness; P, Pupil diameter; Pre-BCVA, Preoperative BCVA, Post-BCVA, Postoperative BCVA, Pre-IOP, Preoperative IOP, Post-IOP, Postoperative IOP; WTW, White-to-White Distance; Model 1 vs. Model 2, χ2 = 13.196, *p* = 0.355; *P*_1, Hyperopic Shift VS. emmetropia, *P*_2, Myopic Shift VS. emmetropia.

WTW was associated with an increased probability of occurrence of HS (Model 1, OR = 2.209, 95% CI: 1.399 to 3.488, *P* = 0.001; Model 2, OR = 2.065, 95% CI: 1.327 to 3.212, *P* = 0.001). Interestingly, post-BCVA was significantly associated with an increased risk of both hyperopic and myopic shifts, yet it showed no correlation with postoperative prediction error. This discrepancy may be due to refractive error contributing to reduced post-BCVA, or alternatively, post-BCVA may be affected by unquantified factors such as the severity of glaucomatous optic neuropathy or coexisting macular pathology. Besides, variables such as BMI, CECD, and IOP had no statistical significance in both univariate and multivariate analysis (*P* > 0.05). These results suggest that AL, LT, and WTW are core factors affecting postoperative refractive outcomes.

### ROC curve analysis

**FIGURE 3:**
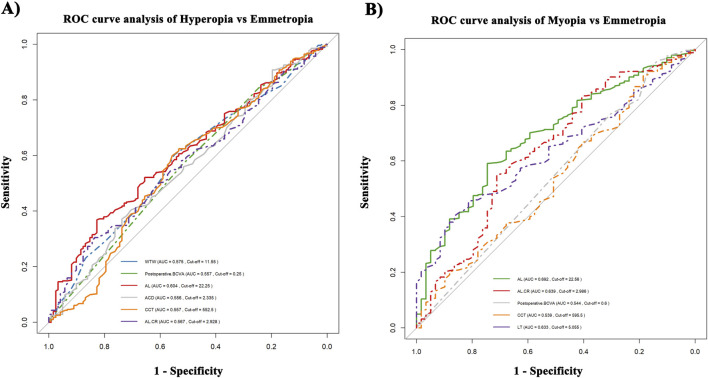
ROC curve analysis. **(A)** Hyperopia vs. Emmetropia. **(B)** Myopia vs. Emmetropia.

Figure 3 demonstrates significant differences in predictive performance between various postoperative refractive outcomes (HS, Emmetropia and MS). AL performed the best in predicting both HS and MS (HS: AUC = 0.604; MS: AUC = 0.692). In contrast, WTW, ACD, CCT, post-BCVA and AL/CR ratio showed poorer performance in distinguishing HS and Emmetropia, with AUC values of 0.575, 0.556, 0.557, 0.557, and 0.567, respectively.

On the other hand, the predictive performance for MS was significantly enhanced, with AL/CR ratio (AUC = 0.639, cut-off value = 2.986) and LT (AUC = 0.633, cut-off value = 5.055 mm). Notably, AL and AL/CR ratio both contribute to predicting MS and HS, but differences in the cut-off values (cut-off for AL, HS: 22.25 mm, MS: 22.56 mm; cut-off for AL/CR, HS: 2.928, MS: 2.986) indicate that the mechanisms of refractive outcome are different.

Moreover, LT was only associated with MS, while WTW was only correlated with HS, indicating that the selection of parameters should vary depending on the type of refractive shift. Overall, the higher AUC values for AL and LT support their significance in refractive prediction models.

## Discussion

This study systematically revealed the heterogeneity in the direction of core anatomical determinants influencing prediction error after PE + IOL surgery in PACG patients, based on retrospective data analysis. The multinomial logistic regression model indicates that AL and LT are independent protective factors for MS, with each 1 mm increase reducing the risk of MS occurrence by approximately 65% (OR = 0.642) and 56.8% (OR = 0.568), respectively. Additionally, each 1 mm increase in WTW is associated with about a 2.2-fold higher risk of HS occurrence (OR = 2.209). ROC curve analysis further identifies risk warning thresholds. Patients with AL > 22.56 mm should be cautious of developing MS postoperatively, while those with AL < 22.25 mm are more likely to develop HS. Moreover, the study found that thicker LT and larger AL/CR ratio are risk factors for MS postoperatively, while smaller WTW and smaller AL/CR ratio are associated with an increased risk of HS occurrence postoperatively. This study highlights that clinical decision-making should integrate the independent effects identified by the multinomial model with the ROC warning thresholds, offering a theoretical basis for the precise, personalized design of surgery in PACG patients.

This study found that the incidence of postoperative refractive errors in PACG patients (44.80%, 181/404) was higher than in conventional cataract patients (8.1%–27.3%) (Lundström et al., 2018; Romero Valero et al., 2022), highlighting the unique impact of anatomical differences on predictive accuracy. The ongoing debate on predicting postoperative refractive error after PE + IOL primarily revolves around the applicability of IOL power calculation formulas, the extent of preoperative AL and ACD changes, and the impact of postoperative IOL position or type on refractive status (Hayashi and Hayashi, 2005; Hirnschall et al., 2013; Ning et al., 2019). A network meta-analysis (Lu et al., 2022) found no significant differences among eight IOL power calculation formulas in PACG patients, suggesting that postoperative refractive status in PACG patients following PE + IOL surgery is not significantly correlated with IOL power calculation formulas. In contrast, previous studies have shown that each 1 mm measurement error in preoperative AL and ACD can result in refractive errors of 2.7D and 1.5D, respectively (Olsen, 2007).

We found that patients with AL < 22.25 mm were more likely to develop HS postoperatively, consistent with previous research. Both the studies by Francis et al. and Li et al. indicated that shorter AL significantly increases the risk of HS occurrence after PE + IOL surgery in PACG patients (Francis et al., 2005; Li et al., 2021). It is important to note that, although multivariate analysis shows that each 1 mm increase in AL reduces the risk of MS occurrence by about 65%, the ROC threshold suggests that patients with AL > 22.56 mm are at higher risk of developing MS postoperatively, possibly due to unaccounted-for posterior displacement of IOL in long axial length patients. Therefore, dynamic assessment of IOL position during surgery should be considered.

In addition to AL, several recent studies have shown that the AL/CR ratio also reflects the refractive status and morphological characteristics of the eye (He et al., 2015; Iyamu et al., 2011; Omoto et al., 2020). It has been demonstrated that PE + IOL surgery causes changes in corneal biomechanical properties (Hirasawa et al., 2018), making this study the first to systematically evaluate the effect of AL/CR ratio on prediction error in PACG patients. Generally, myopic eyes tend to have an AL/CR ratio between 2.9 and 3.1, while emmetropic or hyperopic eyes have a ratio lower than 2.9 (Foo et al., 2016; Scheiman et al., 2016). The optimal AL/CR ratio threshold found in the ROC curve analysis of this study aligns with previous studies: AL/CR ratio >2.986 (AUC = 0.639) indicates a high risk of MS occurrence, with an increase in the ratio possibly caused by a longer AL or smaller corneal radius leading to an underestimation of IOL power and increasing the risk of MS occurrence. Conversely, AL/CR ratio <2.928 (AUC = 0.567) is significantly associated with HS. While AL has a significantly higher independent predictive power for MS or HS than AL/CR, the latter is more useful for evaluating the synergistic effect of AL and cornea, rather than serving as a substitute for a single anatomical characteristic. Figure 3 suggests that forecasting MS may be more dependent on AL, while HS is regulated by multiple factors, including AL, WTW, and corneal curvature. Although the AL/CR ratio shows potential in predictive models, its effect should still be considered alongside other key parameters for comprehensive assessment.

Shin et al. (2023) suggested that preoperative shallow ACD and larger IOP fluctuations are key factors associated with postoperative HS, and these traits are particularly common in ACG eyes. Our findings support this viewpoint, as preoperative shallow ACD was identified as an independent risk factor for postoperative HS. According to ROC analysis, the risk of HS increases significantly when ACD is less than 2.335 mm. Therefore, in PACG patients with shallow ACD, comprehensive preoperative evaluation and vigilant postoperative monitoring of refractive status are strongly recommended. Furthermore, Serdar Bayraktar et al. (Bayraktar et al., 2024) observed that PACG patients have a significant deepening of ACD after PE + IOL compared to conventional cataract patients. Some researchers have proposed that when the IOL deviates to a more posterior position due to ACD deepening or AL shortening, HS is more likely to occur (Francis et al., 2005; Nonaka et al., 2006). This further suggests that the unique ocular anatomy of PACG patients may influence the final position of the IOL, which in turn could cause refractive change.

In addition to AL and ACD, LT and changes in postoperative anatomical structure may also affect the refractive stability of PACG patients. Kang et al. (2009) suggested that PACG patients have a larger lens capsule and looser zonules, which could result in postoperative IOL position instability, subsequently increasing the risk of MS occurrence after surgery. They observed that preoperative thicker LT, along with ACD deepening and AL shortening after PE + IOL, were closely associated with inaccurate IOL power predictions in PACG patients. This study also found that PACG patients with LT > 5.055 mm should be cautious about developing MS postoperatively. WTW, as a variable used in IOL power calculation formulas, has been found in previous studies to increase with AL in patients with AL < 24.5 mm (Wei et al., 2021), which is similarly observed in our study (Figure 3). Additionally, we found that WTW is positively correlated with the risk of HS occurrence in PACG patients. This result suggests that WTW may influence postoperative refractive outcomes through certain mechanisms. It could be that the significant deepening of ACD in PACG patients amplifies the effect of WTW on posterior displacement of IOL, or that the zonular laxity in PACG patients further exacerbates IOL position fluctuations, thereby impacting refractive outcomes. Although some studies indicate that WTW has little effect on postoperative astigmatism after PE + IOL (Zhang et al., 2019), our study found that WTW is an independent risk factor for HS after PE + IOL in PACG patients, and its value should be evaluated preoperatively.

The role of age in postoperative refractive error has also attracted attention. Previous studies have suggested that age may influence the accuracy of IOL power calculation following PE + IOL using the SRK/T formula (Hayashi et al., 2016). Furthermore, as age increases, it can affect the anatomical structures of the anterior segment, including the Schlemm’s canal, trabecular meshwork morphology, and the accuracy of ACD measurements (Chen et al., 2018). However, Li et al. found that the actual effect of age is small, and therefore, age was not considered a significant predictor of postoperative refractive error (Li et al., 2021). Our findings also confirm this, as although age was significantly positively correlated with prediction error, multivariate analysis did not reveal a clear relationship between age and postoperative refractive status. Therefore, we conclude that age is not a significant determinant of postoperative prediction error.

In addition to the ocular biometric parameters, the impact of a history of previous glaucoma surgery and other combined surgical approaches on postoperative refractive error in PACG patients remains to be further explored. Zhang et al. (2013) found that in patients who had previously undergone trabeculectomy, postoperative refractive error following PE + IOL was significantly correlated with postoperative IOP changes. Increased postoperative IOP was associated with MS, while decreased IOP was associated with HS. In contrast, this study did not observe significant effects of preoperative IOP, postoperative IOP, or ∆IOP on postoperative refractive error. This may be related to significant changes in postoperative corneal morphology caused by trabeculectomy (e.g., increased corneal curvature, shortened axial length, and shallower anterior chamber). Furthermore, although patients with high preoperative IOP had elevated corneal posterior surface, the change in CCT was small (Koivusalo and Välimäki, 2020), which may explain why ∆IOP did not significantly affect postoperative refractive error in this study.

Interestingly, in PACG patients undergoing PE + IOL combined with trabeculectomy, postoperative AL also shortens to varying extents, with the shortening being more significant than in PE + IOL patients (Mehta et al., 2022). Additionally, the degree of shortening correlates positively with the extent of postoperative IOP reduction (Francis et al., 2005). These changes may lead to IOL power calculation deviations, as traditional formulas based on preoperative biometric parameters cannot dynamically predict postoperative axial length changes. Recently, a randomized clinical trial by Xiulan Zhang’s team (Song et al., 2025) demonstrated that PE + IOL combined with GSL in PACG patients with cataracts had an IOP-lowering effect comparable to that of PE + IOL combined with trabeculectomy. In this study, postoperative refractive error did not significantly increase in patients who underwent PE + IOL combined with GSL, indicating that GSL causes minimal interference with IOL positioning and refractive prediction while maintaining anterior chamber angle openness. This finding supports GSL as the preferred combined treatment option for PACG patients, offering both advantages in IOP control and refractive stability.

The effects of other combined surgical approaches are more complex. Wang et al. (2016) emphasized that PE + IOL combined with endoscopic cyclophotocoagulation (ECP) can cause ciliary body contraction, leading to changes in zonular tension, which results in anterior displacement of the IOL and lens capsule complex, thereby causing postoperative refractive status toward myopia. Furthermore, several studies (Francis et al., 2005; Hammel et al., 2013; Ibarz Barberá et al., 2021) have found that glaucoma drainage device surgery can also cause slight changes in early postoperative corneal astigmatism, AL, and ACD, but these changes stabilize after 3 months. Although this study found no significant association between combined other glaucoma surgeries and postoperative refractive error (*χ* ^*2*^ = 4.699, *P* = 0.095), it suggests that the potential risks of specific surgeries, particularly ECP and those that may alter ciliary body anatomical relationships, should be carefully considered.

For patients with zonular instability, PE + IOL can be combined with CTR implantation, but the impact of CTR implantation on refractive status must also be carefully evaluated. Park et al. (2016) found that after PE + IOL combined with CTR implantation, the posterior displacement of IOL could lead to postoperative refractive status toward hyperopia. Furthermore, a meta-analysis by Xu et al. (2025) further confirmed that CTR implantation is associated with an increased risk of HS occurrence. In this study, we found that among patients who underwent PE + IOL combined with capsular tension ring, the proportion of HS was significantly higher (Z value = +2.95), which may be related to the posterior displacement of IOL after expansion of capsular bag. However, the effect of CTR in the multinomial model did not reach the significance threshold (*P_1* = 0.276, *P_2* = 0.150), suggesting that the sample size should be expanded to clarify its clinical significance.

The core strength of this study lies in its systematic assessment of the multidimensional factors influencing postoperative refractive error in PACG patients after PE + IOL surgery, incorporating multinomial regression models and ROC curve analysis. It provides quantifiable risk warning thresholds to assist clinicians in making informed decisions. AL > 22.56 mm serves as a key early warning indicator for postoperative MS. For patients with AL > 22.56 mm, especially when LT > 5.055 mm and AL/CR ratio >2.986, we recommend using more accurate IOL calculation formulas, such as the Barrett TK, along with a reserved hyperopic offset of +0.50D to mitigate the risk of postoperative myopic drift. Frequent postoperative refraction monitoring is also advised to facilitate early detection and management of potential refractive instability. The risk of HS is primarily driven by the combined influence of WTW and AL. In patients with WTW <11.55 mm and AL < 22.25 mm, a myopic offset of −0.50D is advisable, together with preferential use of low-spherical-aberration aspheric IOLs to enhance postoperative visual quality. Additionally, post-BCVA <0.25 may serve as an early indicator of HS. Dynamic BCVA monitoring should be interpreted in conjunction with other biometric parameters to improve the accuracy of refractive stability predictions. The study further highlights the heterogeneous effects of different combined surgical approaches on refractive stability, such as the advantage of GSL in maintaining refractive prediction accuracy and the potential association between CTR implantation and HS.

However, this study has certain limitations. First, the sample size was insufficient, and the follow-up period was relatively short, which may limit the statistical power of evaluating certain variables. Secondly, the study did not include postoperative dynamic biometric parameters (e.g., real-time IOL position monitoring, corneal biomechanical changes), nor did it collect core indicators of postoperative glaucoma progression. Therefore, it was not possible to clarify the potential association between postoperative prediction error and structural or functional changes related to glaucoma, which may have led to the omission of key underlying mechanisms. Third, although multinomial logistic regression analyses revealed the independent predictive value of variables such as AL, LT, and WTW, their corresponding AUC values generally ranged lower than 0.7, indicating only mild to moderate discriminatory power. This suggests that their predictive effectiveness as standalone indicators is limited. Therefore, in clinical practice, these variables are more appropriately used as risk warning tools in combination with other parameters for comprehensive preoperative assessment. In future studies, we plan to increase the sample size and extend the follow-up period, focusing on tracking refractive stability changes for more than 1 year postoperatively. Additionally, we will integrate intraoperative OCT technology for dynamic monitoring of IOL position and corneal morphology, coupled with machine learning algorithms to develop adaptive refractive prediction models.

## Conclusion

This study systematically evaluated the multidimensional factors influencing postoperative refractive error in PACG patients with PE + IOL. AL, LT, and WTW are the core predictive indicators for postoperative refractive outcomes. AL > 22.56 mm, LT > 5.055 mm, or AL/CR > 2.986 indicate a high risk of MS occurrence, while AL < 22.25 mm, WTW <11.55 mm, or AL/CR < 2.928 are significantly associated with HS. Among patients who underwent PE + IOL combined with capsular tension ring, the proportion of HS was significantly higher (Z value = +2.95). This study provides a theoretical foundation for optimizing postoperative refractive outcomes in PACG patients, recommending more precise combined surgical options that balance the need for refractive stability and IOP control.

## Data Availability

The original contributions presented in the study are included in the article/Supplementary Material, further inquiries can be directed to the corresponding authors.

## References

[B1] Angmo D. Shakrawal J. Gupta B. Yadav S. Pandey R. M. Dada T. (2019). Comparative evaluation of phacoemulsification alone *versus* phacoemulsification with goniosynechialysis in primary angle-closure glaucoma: a randomized controlled trial. Ophthalmol. Glaucoma 2 (5), 346–356. 10.1016/j.ogla.2019.05.004 32672677

[B2] Bayraktar S. Yıldırım Erdal B. D. Altaş F. B. Türkay M. Şen E. (2024). The effects of lens extraction surgery on intraocular pressure and anterior segment parameters in primary angle-closure glaucoma. Turk J. Ophthalmol. 54 (1), 32–37. 10.4274/tjo.galenos.2023.82453 38385318 PMC10895158

[B3] Chen Z. Sun J. Li M. Liu S. Chen L. Jing S. (2018). Effect of age on the morphologies of the human Schlemm's canal and trabecular meshwork measured with swept-source optical coherence tomography. Eye (Lond) 32 (10), 1621–1628. 10.1038/s41433-018-0148-6 29921951 PMC6189106

[B4] Day A. C. Cooper D. Burr J. Foster P. J. Friedman D. S. Gazzard G. (2018). Clear lens extraction for the management of primary angle closure glaucoma: surgical technique and refractive outcomes in the EAGLE cohort. Br. J. Ophthalmol. 102 (12), 1658–1662. 10.1136/bjophthalmol-2017-311447 29453222

[B5] Fang Z. Song Y. Jin L. Han Y. Zhang X. (2025). Phacoemulsification combined with trabecular meshwork-Schlemm canal-based minimally invasive glaucoma surgery in primary angle-closure glaucoma: a systematic review and meta-analysis. BMC Ophthalmol. 25 (1), 168. 10.1186/s12886-025-04005-y 40181304 PMC11966876

[B6] Foo V. H. Verkicharla P. K. Ikram M. K. Chua S. Y. Cai S. On Behalf Of The Gusto Study, G (2016). Axial length/corneal radius of curvature ratio and myopia in 3-Year-Old children. Transl. Vis. Sci. Technol. 5 (1), 5. 10.1167/tvst.5.1.5 PMC4757459 26929885

[B7] Foster P. J. Buhrmann R. Quigley H. A. Johnson G. J. (2002). The definition and classification of glaucoma in prevalence surveys. Br. J. Ophthalmol. 86 (2), 238–242. 10.1136/bjo.86.2.238 11815354 PMC1771026

[B8] Francis B. A. Wang M. Lei H. Du L. T. Minckler D. S. Green R. L. (2005). Changes in axial length following trabeculectomy and glaucoma drainage device surgery. Br. J. Ophthalmol. 89 (1), 17–20. 10.1136/bjo.2004.043950 15615739 PMC1772469

[B9] George R. Panda S. Vijaya L. (2022). Blindness in glaucoma: primary open-angle glaucoma versus primary angle-closure glaucoma-a meta-analysis. Eye (Lond) 36 (11), 2099–2105. 10.1038/s41433-021-01802-9 34645961 PMC9582001

[B10] Ghadamzadeh M. Karimi F. Ghasemi Moghaddam S. Daneshvar R. (2022). Anterior chamber angle changes in primary angle-closure glaucoma following phacoemulsification Versus phacotrabeculectomy: a prospective randomized clinical trial. J. Glaucoma 31 (3), 147–155. 10.1097/ijg.0000000000001977 35210384

[B11] Hammel N. Lusky M. Kaiserman I. Robinson A. Bahar I. (2013). Changes in anterior segment parameters after insertion of Ex-PRESS miniature glaucoma implant. J. Glaucoma 22 (7), 565–568. 10.1097/IJG.0b013e31824d4fa1 22407394

[B12] Hayashi K. Hayashi H. (2005). Comparison of the stability of 1-piece and 3-piece acrylic intraocular lenses in the lens capsule. J. Cataract. Refract Surg. 31 (2), 337–342. 10.1016/j.jcrs.2004.06.042 15767155

[B13] Hayashi K. Ogawa S. Yoshida M. Yoshimura K. (2016). Influence of patient age on intraocular lens power prediction error. Am. J. Ophthalmol. 170, 232–237. 10.1016/j.ajo.2016.08.016 27562431

[B14] He M. Foster P. J. Ge J. Huang W. Zheng Y. Friedman D. S. (2006). Prevalence and clinical characteristics of glaucoma in adult Chinese: a population-based study in liwan district, Guangzhou. Invest Ophthalmol. Vis. Sci. 47 (7), 2782–2788. 10.1167/iovs.06-0051 16799014

[B15] He X. Zou H. Lu L. Zhao R. Zhao H. Li Q. (2015). Axial length/corneal radius ratio: association with refractive state and role on myopia detection combined with visual acuity in Chinese schoolchildren. PLoS One 10 (2), e0111766. 10.1371/journal.pone.0111766 25693186 PMC4333577

[B16] Hirasawa K. Nakakura S. Nakao Y. Fujino Y. Matsuura M. Murata H. (2018). Changes in corneal biomechanics and intraocular pressure following cataract surgery. Am. J. Ophthalmol. 195, 26–35. 10.1016/j.ajo.2018.07.025 30071213

[B17] Hirnschall N. Amir-Asgari S. Maedel S. Findl O. (2013). Predicting the postoperative intraocular lens position using continuous intraoperative optical coherence tomography measurements. Invest Ophthalmol. Vis. Sci. 54 (8), 5196–5203. 10.1167/iovs.13-11991 23761092

[B18] Ibarz Barberá M. Morales-Fernandez L. Gómez de Liaño R. Tañá Rivero P. Teus M. A. (2021). Changes to corneal topography and biometrics after PRESERFLO microshunt surgery for glaucoma. J. Glaucoma 30 (10), 921–931. 10.1097/ijg.0000000000001912 34255755

[B19] Issa S. Ginés-Gallego C. Griffin B. Dervenis P. Dimitriou C. Pavel M. (2025). Cataract surgery with and without trabecular micro-bypass stent in primary angle-closure glaucoma: a multi-centre cohort study. Eye (Lond) 10.1038/s41433-025-03923-x PMC12446436 40691726

[B20] Iyamu E. Iyamu J. Obiakor C. I. (2011). The role of axial length-corneal radius of curvature ratio in refractive state categorization in a nigerian population. ISRN Ophthalmol. 2011, 138941. 10.5402/2011/138941 24527225 PMC3912816

[B21] Ji Z. Ma J. Sun Z. Zhang M. (2024). Comparison of two formulas for calculating intraocular lens in patients with angle-closure glaucoma. Med. Baltim. 103 (45), e40387. 10.1097/md.0000000000040387 PMC11556955 39533616

[B22] Kang S. Y. Hong S. Won J. B. Seong G. J. Kim C. Y. (2009). Inaccuracy of intraocular lens power prediction for cataract surgery in angle-closure glaucoma. Yonsei Med. J. 50 (2), 206–210. 10.3349/ymj.2009.50.2.206 19430552 PMC2678694

[B23] Koivusalo R. Välimäki J. (2020). Effect of glaucoma drainage implant surgery on corneal topography: a prospective study. Acta Ophthalmol. 98 (3), 305–309. 10.1111/aos.14247 31495070

[B24] Li Y. Guo C. Huang C. Jing L. Huang Y. Zhou R. (2021). Development and evaluation of the prognostic nomogram to predict refractive error in patients with primary angle-closure glaucoma who underwent cataract surgery combined with goniosynechialysis. Front. Med. (Lausanne) 8, 749903. 10.3389/fmed.2021.749903 34977061 PMC8714900

[B25] Lu W. Hou Y. Yang H. Sun X. (2022). A systemic review and network meta-analysis of accuracy of intraocular lens power calculation formulas in primary angle-closure conditions. PLoS One 17 (10), e0276286. 10.1371/journal.pone.0276286 36240196 PMC9565378

[B26] Lundström M. Dickman M. Henry Y. Manning S. Rosen P. Tassignon M. J. (2018). Risk factors for refractive error after cataract surgery: analysis of 282 811 cataract extractions reported to the european registry of quality outcomes for cataract and refractive surgery. J. Cataract. Refract Surg. 44 (4), 447–452. 10.1016/j.jcrs.2018.01.031 29685779

[B27] Mehta R. Tomatzu S. Cao D. Pleet A. Mokhur A. Aref A. A. (2022). Refractive outcomes for combined phacoemulsification and glaucoma drainage procedure. Ophthalmol. Ther. 11 (1), 311–320. 10.1007/s40123-021-00434-2 34870803 PMC8770753

[B28] Ning X. Yang Y. Yan H. Zhang J. (2019). Anterior chamber depth - a predictor of refractive outcomes after age-related cataract surgery. BMC Ophthalmol. 19 (1), 134. 10.1186/s12886-019-1144-8 31238910 PMC6591866

[B29] Nonaka A. Kondo T. Kikuchi M. Yamashiro K. Fujihara M. Iwawaki T. (2006). Angle widening and alteration of ciliary process configuration after cataract surgery for primary angle closure. Ophthalmology 113 (3), 437–441. 10.1016/j.ophtha.2005.11.018 16513457

[B30] Olsen T. (2007). Calculation of intraocular lens power: a review. Acta Ophthalmol. Scand. 85 (5), 472–485. 10.1111/j.1600-0420.2007.00879.x 17403024

[B31] Omoto M. K. Torii H. Hayashi K. Ayaki M. Tsubota K. Negishi K. (2020). Ratio of axial length to corneal radius in Japanese patients and accuracy of intraocular lens power calculation based on biometric data. Am. J. Ophthalmol. 218, 320–329. 10.1016/j.ajo.2020.03.006 32209342

[B32] Park H. J. Lee H. Kim do W. Kim E. K. Seo K. Y. Kim T. I. (2016). Effect of Co-Implantation of a capsular tension ring on clinical outcomes after cataract surgery with monofocal intraocular lens implantation. Yonsei Med. J. 57 (5), 1236–1242. 10.3349/ymj.2016.57.5.1236 27401657 PMC4960392

[B33] Romero Valero D. Escolano Serrano J. Monera Lucas C. E. Castilla Martínez G. Martínez Toldos J. J. (2022). Limits of the precision in refractive results after cataract surgery. Arch. Soc. Esp. Oftalmol. Engl. Ed. 97 (7), 370–375. 10.1016/j.oftale.2021.11.002 35624062

[B34] Scheiman M. Gwiazda J. Zhang Q. Deng L. Fern K. Manny R. E. (2016). Longitudinal changes in corneal curvature and its relationship to axial length in the correction of Myopia evaluation trial (COMET) cohort. J. Optom. 9 (1), 13–21. 10.1016/j.optom.2015.10.003 26564446 PMC4705324

[B35] Shan S. Wu J. Cao J. Feng Y. Zhou J. Luo Z. (2024). Global incidence and risk factors for glaucoma: a systematic review and meta-analysis of prospective studies. J. Glob. Health 14, 04252. 10.7189/jogh.14.04252 39513294 PMC11544525

[B36] Shin J. H. Kim S. H. Oh S. Lee K. M. (2023). Factors associated with refractive prediction error after phacotrabeculectomy. J. Clin. Med. 12 (17), 5706. 10.3390/jcm12175706 37685774 PMC10488334

[B37] Song Y. Fan S. Tang L. Lin F. Li F. Lv A. (2025). Two-year outcomes of phacogoniotomy vs phacotrabeculectomy for advanced primary angle-closure glaucoma with cataract: a noninferiority randomized clinical trial. JAMA Ophthalmol. 143, 462–469. 10.1001/jamaophthalmol.2025.0685 40244620 PMC12006911

[B38] Song Y. Xie L. Zhu X. Fan S. Lv A. Tang G. (2025). Two-year outcome of phacogoniotomy for advanced primary angle-closure glaucoma with cataracts: a multicentre study. Br. J. Ophthalmol. 109 (7), 770–774. 10.1136/bjo-2024-325375 40049755

[B39] Tham Y. C. Li X. Wong T. Y. Quigley H. A. Aung T. Cheng C. Y. (2014). Global prevalence of glaucoma and projections of glaucoma burden through 2040: a systematic review and meta-analysis. Ophthalmology 121 (11), 2081–2090. 10.1016/j.ophtha.2014.05.013 24974815

[B40] Wang J. C. Campos-Möller X. Shah M. Sheybani A. Ahmed I. I. (2016). Effect of endocyclophotocoagulation on refractive outcomes in angle-closure eyes after phacoemulsification and posterior chamber intraocular lens implantation. J. Cataract. Refract Surg. 42 (1), 132–137. 10.1016/j.jcrs.2015.07.046 26948788

[B41] Wei L. He W. Meng J. Qian D. Lu Y. Zhu X. (2021). Evaluation of the white-to-white distance in 39,986 Chinese cataractous eyes. Invest Ophthalmol. Vis. Sci. 62 (1), 7. 10.1167/iovs.62.1.7 PMC7794278 33393973

[B42] Xu B. Y. Richter G. M. Burkemper B. S. Wang D. Jiang X. Torres M. (2025). Prevalence and risk factors of primary angle closure disease in an adult Chinese American population: the Chinese American eye study. Am. J. Ophthalmol. 274, 32–41. 10.1016/j.ajo.2025.02.037 40023353 PMC12043426

[B43] Xu S. Zhang Y. Li X. Si W. Tian G. Yang Y. (2025). Effect of implanted capsular tension ring on postoperative refractive shift: a systematic review and meta-analysis. Semin. Ophthalmol. 40 (3), 162–168. 10.1080/08820538.2024.2381770 39039754

[B44] Zaidi F. H. Corbett M. C. Burton B. J. Bloom P. A. (2007). Raising the benchmark for the 21st century--the 1000 cataract operations audit and survey: outcomes, consultant-supervised training and sourcing NHS choice. Br. J. Ophthalmol. 91 (6), 731–736. 10.1136/bjo.2006.104216 17050577 PMC1955623

[B45] Zhang N. Tsai P. L. Catoira-Boyle Y. P. Morgan L. S. Hoop J. S. Cantor L. B. (2013). The effect of prior trabeculectomy on refractive outcomes of cataract surgery. Am. J. Ophthalmol. 155 (5), 858–863. 10.1016/j.ajo.2012.11.023 23398980

[B46] Zhang H. Tang G. Liu J. (2016). Effects of phacoemulsification combined with goniosynechialysis on primary angle-closure glaucoma. J. Glaucoma 25 (5), e499–e503. 10.1097/ijg.0000000000000297 26066498

[B47] Zhang W. Pasricha N. D. Kuo A. N. Vann R. R. (2019). Influence of corneal diameter on surgically induced astigmatism in small-incision cataract surgery. Can. J. Ophthalmol. 54 (5), 556–559. 10.1016/j.jcjo.2018.12.013 31564344

