# Supplementary material for: Refractive error after phacoemulsification combined with intraocular lens implantation in primary angle-closure glaucoma: a multifactorial analysis of biometric parameters and surgical strategies
Source: Front Cell Dev Biol. 2025 Aug 22;13:1654719. doi: 10.3389/fcell.2025.1654719 (PMC12411433; doi:10.3389/fcell.2025.1654719)
Supplement: Supplementary file 1 [file Table1.docx]

**Supplemental table 1** Comparison of All Models

| **Model** | **Variables Included** | **AIC** | **Likelihood Ratio χ²** | ***P*** |
| --- | --- | --- | --- | --- |
| **Model 1**  **(Full model)** | Age, ACD, AL, AL/CR,  CCT, LT, Sex, WTW, Diabetes,  Preoperative BCVA, Postoperative BCVA,  Combined with CTR implantation, Combined with other glaucoma surgery | 747.459 | 13.196 | 0.355 |
| **Model 2**  **(Optimal model from stepwise)** | ACD, AL, CCT, LT, WTW, Postoperative BCVA, Combined with CTR implantation | 736.655 |  |  |
